# Sex-specific epigenetic development in the mouse hypothalamic arcuate nucleus pinpoints human genomic regions associated with body mass index

**DOI:** 10.1126/sciadv.abo3991

**Published:** 2022-09-28

**Authors:** Harry MacKay, Chathura J. Gunasekara, Kit-Yi Yam, Dollada Srisai, Hari Krishna Yalamanchili, Yumei Li, Rui Chen, Cristian Coarfa, Robert A. Waterland

**Affiliations:** ^1^ USDA/ARS Children’s Nutrition Research Center, Department of Pediatrics, Baylor College of Medicine, Houston, TX, USA.; ^2^ Department of Molecular Physiology and Biophysics, Vanderbilt University, Nashville, TN, USA.; ^3^ Department of Pediatrics, Baylor College of Medicine, Houston, TX, USA.; ^4^ Jan and Dan Duncan Neurological Research Institute, Texas Children’s Hospital, Houston, TX, USA.; ^5^ Human Genome Sequencing Center, Department of Molecular and Human Genetics, Baylor College of Medicine, Houston, TX, USA.; ^6^ Department of Molecular and Cell Biology, Baylor College of Medicine, Houston, TX, USA.; ^7^ Dan L. Duncan Cancer Center, Baylor College of Medicine, Houston, TX, USA.; ^8^ Department of Molecular and Human Genetics, Baylor College of Medicine, Houston, TX, USA.

## Abstract

Recent genome-wide association studies corroborate classical research on developmental programming indicating that obesity is primarily a neurodevelopmental disease strongly influenced by nutrition during critical ontogenic windows. Epigenetic mechanisms regulate neurodevelopment; however, little is known about their role in establishing and maintaining the brain’s energy balance circuitry. We generated neuron and glia methylomes and transcriptomes from male and female mouse hypothalamic arcuate nucleus, a key site for energy balance regulation, at time points spanning the closure of an established critical window for developmental programming of obesity risk. We find that postnatal epigenetic maturation is markedly cell type and sex specific and occurs in genomic regions enriched for heritability of body mass index in humans. Our results offer a potential explanation for both the limited ontogenic windows for and sex differences in sensitivity to developmental programming of obesity and provide a rich resource for epigenetic analyses of developmental programming of energy balance.

## Teaser

Obesity risk may be largely determined by epigenetic development of the brain during early life.

## INTRODUCTION

The global prevalence of obesity has increased rapidly in recent decades to affect more than 2 billion people, making it one of the largest contributors to poor health worldwide (*1*). Classic studies in both rodents (*2*) and humans (*3*) show that nutrition during critical early-life periods can permanently alter energy balance regulation, predisposing to obesity (*4*–*7*). Such phenomena are known as developmental programming. More recently, large genome-wide association studies (GWAS) have corroborated a developmental origin for obesity by discovering that GWAS variants associated with adult body mass index (BMI: kilograms per square meter) are strongly enriched for neurodevelopmental genes (*8*, *9*) and chromatin marks in fetal brain (*10*). Accordingly, and consistent with previous reports (*11*, *12*), we suggest that it may be useful to consider obesity as a neurodevelopmental disease. This proposal is supported by recent data on maternal obesity; currently, one-third of all reproductive-age women in the United States are obese (BMI ≥ 30) (*13*). Extensive human (*14*) and animal model data (*15*, *16*) indicate that maternal obesity during pregnancy and/or lactation promotes obesity in her offspring, potentially leading to transgenerational amplification of obesity. Maternal obesity additionally appears to increase the risk of neurodevelopmental outcomes including attention deficit hyperactivity disorder and autism spectrum disorder (*13*, *17*). The increasing prevalence of maternal obesity in the developed world in recent decades is mirrored by increases in the prevalence of these disorders (*18*, *19*), suggesting that the establishment of energy balance regulation is one of several neurodevelopmental outcomes that are adversely affected by maternal obesity.

Like obesity, neural tube defects including spina bifida and anencephaly are developmental outcomes that are influenced both by genetics and by nutrition during a critical ontogenic period. The success of population-level intervention to prevent neural tube defects suggests a model for primary prevention of obesity. In particular, a link with dietary folate had been known for decades, but the discovery that neural tube closure is an early embryonic event made it clear that prevention would require dietary fortification at the population level (*20*). Analogously, because neurodevelopment is intimately dependent on epigenetic mechanisms (*21*–*23*), we propose that effective interventions to prevent obesity may depend on a detailed understanding of developmental epigenetics within the hypothalamus and other brain regions. During developmental periods when epigenetic mechanisms are undergoing establishment or maturation, transient environmental influences can induce persistent epigenetic changes (*24*, *25*). Epigenetic mechanisms may therefore account for two hallmarks of developmental programming of energy balance: plasticity limited to specific developmental periods of (i.e., critical windows) (*24*) and sex-specific effects (*26*). Cytosine methylation (5mC) and hydroxymethylation (5hmC) constitute the two main epigenetic modifications of DNA in the brain, with relatively high levels of 5hmC being a unique feature of brain tissue (*27*). With this in mind, we hypothesized that sex-specific postnatal epigenetic maturation in the hypothalamic arcuate nucleus (ARH), a key node in the energy balance circuitry, defines the ontogeny of the early postnatal critical window (*2*) for developmental programming of energy balance regulation and obesity risk. Because previous studies of hypothalamic developmental epigenetics (*28*–*31*) lack the temporal-, regional-, cell type–, and/or sex-specific resolution required to evaluate this question, we used unbiased genome-wide approaches to screen for sex-specific epigenetic development in postnatal ARH neurons and glia. Our results document marked sex differences in epigenetic maturation in the ARH, supporting our hypothesis. Unexpectedly, we find that human orthologs of murine genomic regions undergoing sex-specific epigenetic maturation in ARH neurons are enriched for genetic variants associated with BMI, implicating developmental epigenetics at the nexus of genetic and early environmental risk factors for obesity.

## RESULTS

### Sex-specific epigenetic development in ARH neurons

**Fig. 1.**
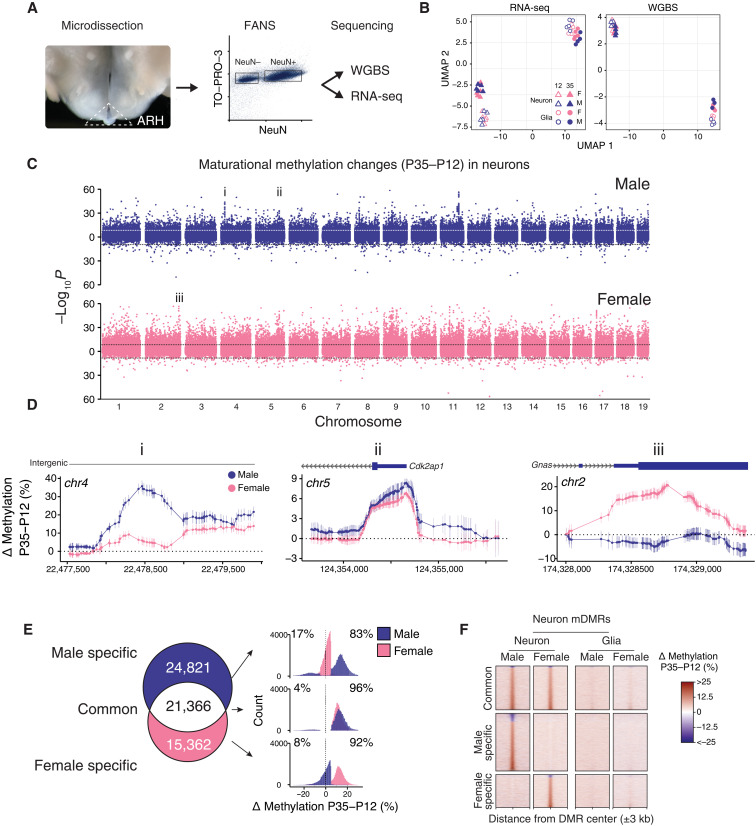
Postnatal epigenetic maturation in ARH neurons is sex specific. (**A**) Illustration of ARH microdissection approach (left) and representative scatterplot from FANS (right). (**B**) UMAP clustering of autosomal RNA-seq and WGBS data on ARH neurons and glia from both ages and sexes. (**C**) Miami plot showing the significance of P35 to P12 differential methylation, at the CpG level, in male and female neurons. Points plotted above/below the origin represent CpGs that gained/lost methylation from P35 to P12, respectively. *Y* axis represents raw *P* values. Genome-wide significance threshold (horizontal dotted lines) = *P* < 2.45 × 10^−9^. (**D**) CpG-level plots of example regions from (C) showing clusters of male-specific (i), common (ii), and female-specific (iii) differentially methylated CpGs. (**E**) Venn diagram illustrating the overlap of mDMRs identified in male and female ARH neurons. Histograms (right) show the magnitudes and directions of these maturational methylation changes. Median differential methylation for P35 > P12 mDMRs ranged from 12.3 to 15.3%, and 13.8 to 17.5% in P35 < P12 mDMRs. (**F**) Heatmaps showing P35 to P12 differential methylation in 6-kb genomic regions centered on neuron mDMRs; most do not show methylation changes in glia.

If the postnatal day 21 (P21) closure of the critical window for developmental programming of energy balance regulation in rodents (*2*) is dictated by epigenetic maturation in the ARH, important epigenetic development in ARH neurons and/or glia must occur around this time. Accordingly, at ages flanking P21 (P12 and P35), we used microdissection and fluorescence-activated nuclear sorting (FANS) followed by whole-genome bisulfite sequencing (WGBS) and RNA sequencing (RNA-seq) to comprehensively profile changes in DNA methylation/hydroxymethylation and gene expression in ARH neurons and glia of male and female mice (*n* = 3 to 5 per cell type, age, and sex combination, with a total of *N* = 34 libraries for WGBS and *N* = 39 libraries for RNA-seq; table S1). Because WGBS does not distinguish between 5mC and 5hmC, we will refer to steady-state measures as methylation/hydroxymethylation but continue to refer to comparative measures as differential methylation (5mC) to simplify the text, acknowledging that changes in methylation may stabilize as either of the two marks. Our deep WGBS data achieve an average autosomal per-CpG read depth of 77× within each cell type, age, and sex group (Fig. 1A; fig. S1, A and B; and table S1). The differentially methylated regions (DMRs) that we detected between neurons and glia in the ARH overlap substantially with those detected in the mouse cortex (fig. S1C) (*23*). In addition, of the seven neuron-versus-glia DMRs that we previously validated by pyrosequencing in the cortex (*32*), a total of six (86%) were also detected in the ARH (χ^2^ = 9.55, *P* < 0.01), supporting the reliability of our WGBS data. Consistent with observations in the cerebral cortex (*23*), average autosome-wide CpH methylation/hydroxymethylation increased from P12 to P35 in ARH neurons; average autosome-wide CpG methylation/hydroxymethylation did not change (fig. S1D). We focused our analysis on methylation/hydroxymethylation within the CpG context because of its enrichment in regulatory regions (*23*) and amenability to read-level methylation analysis (*33*). Unsupervised clustering of genome-wide transcriptomic and CpG methylation/hydroxymethylation data (Fig. 1B) grouped samples primarily by cell type; transcriptomic data on ARH neurons additionally segregated by age. Of the 18,996,589 autosomal CpGs covered, more than 25% were differentially methylated between neurons and glia; just more than 1% showed effects of age and sex, respectively (fig. S1E). In both ARH neurons (Fig. 1, C and D) and glia (fig. S1F), however, many maturational changes from P12 to P35 were sex specific.

In fact, when we integrated across proximal CpGs to identify maturational DMRs (mDMRs), most (65.3 and 82.1%) were sex specific in neurons (Fig. 1, E and F; and table S2) and glia (figs. S1G and S2A), respectively. We identified mDMRs showing a minimum absolute methylation difference of 5% and false discovery rate (FDR) < 0.05. Consistent with the CpG-level analysis (Fig. 1C), most mDMRs show methylation increases, of median range of 12 to 15% (Fig. 1E). We asked to what extent sex-specific mDMRs reflect different developmental trajectories. In neurons, at 40.5% of male-specific mDMRs, methylation in females had already reached the mature level by P12 and was stable thereafter (fig. S2B); we term these “female-precocious mDMRs.” Among female-specific DMRs, on the other hand, only 14.8% were male-precocious (fig. S2C). This indicates that a substantial component of epigenetic maturation in ARH neurons is completed earlier in females than in males. In glia, epigenetic maturation was largely distinct from that in neurons (fig. S2A) but likewise showed extensive sexual dimorphism and a bias toward female precocity (38.5 versus 22.2%, respectively; fig. S2, D and E).

**Fig. 2.**
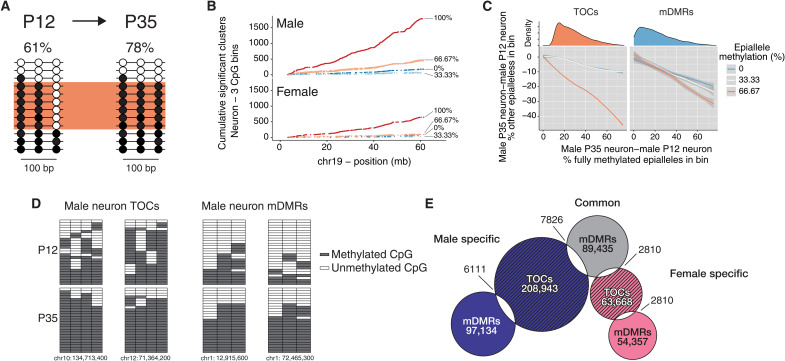
Read-level analysis indicates epigenetic maturation in subsets of ARH neurons. (**A**) Methylation changes that occur in only a subset of ARH neurons (orange box) may not be detected by conventional DMR callers; at each age, average methylation across all reads is shown. (**B**) Cumulative frequency along chromosome 19 of epiallele methylation states enriched in P35 relative to P12 neurons. Data are shown for three-CpG genomic bins by bin average methylation (0%: 000; 33.3%: 100, 010, or 001; 66.7%: 101, 110, or 011; 100%: 111; unmethylated/methylated CpGs represented by 0/1). The dominant feature is the enrichment of new 100% methylated clusters. (**C**) For such regions (left), proportional losses of partially methylated clusters (*y* axis) are plotted versus corresponding gains of fully methylated clusters at P35 relative to P12. The steep orange line indicates that most increases in fully methylated clusters arise from 66.7% methylated clusters (i.e., just one additional CpG site methylated); we call these TOCs. By contrast, at mDMRs (right), gains in fully methylated clusters arise equally from all other epialleles. Density distributions (top) show that most TOCs involve fewer than 40% of the reads in each bin. (**D**) Plots of read-level methylation and epiallele proportions in representative male neuronal TOC and mDMR bins. In the grids, rows and columns represent sequence reads and CpG sites, respectively. (**E**) Venn diagram illustrates numbers and overlaps of TOCs and mDMRs identified in male and female ARH neurons.

Whereas we did study neurons and glia separately, ARH neurons are themselves heterogeneous, consisting of subpopulations defined by the expression of *Agrp*, *Pomc*, *Kiss1*, *Ghrh*, and others (*34*). Epigenetic maturation occurring only in ARH neuronal subtypes (Fig. 2A) may not be detected by conventional DMR callers. We therefore reanalyzed our WGBS data using cluster-based analysis of CpG methylation (CluBCpG), which partitions the genome into 100–base pair (bp) bins to detect clusters of read-level methylation patterns representing different cell types (*33*). By far, the predominant cluster-level change in ARH neurons from P12 to P35 was the formation of fully methylated (100%) read clusters (Fig. 2B), which occurred more frequently in males than in females. Results in glia (fig. S3A) were similar (table S3). In contrast to mDMR bins, in which methylation changes occur at any number of CpGs in the bin, those enriched for fully methylated reads tended to gain methylation at just one CpG site in an epiallele cluster, bringing it to full methylation (Fig. 2, C and D, and fig. S3, B to D); we therefore term these “topped-off clusters” (TOCs) (Fig. 2D). TOCs exhibited higher rates of sex specificity than mDMRs (88.86 and 92.4% in neurons and glia, respectively). Accordingly, we focused our analysis on sex-specific TOCs. These show little overlap with mDMRs (Fig. 2E) and very different associations with genic features. Specifically, whereas neuronal mDMRs are strongly associated with CpG islands (CGIs) upstream and downstream of genes, TOCs are depleted within CGIs but enriched in all non-CGI genic contexts (fig. S3E). Our interpretation is that TOCs represent genomic regions targeted for postnatal maturational “topping off” of methylation within specific ARH neuronal and glial subtypes.

We next asked to what extent maturational methylation changes affect gene expression over this same postnatal epoch. Compared to the 9968 differentially expressed genes (DEGs) between neurons and glia, there were only 863 maturational DEGs (P35 versus P12) in neurons and far fewer sex DEGs (fig. S4, A to C, and table S4). Unlike cell type–specific methylation differences near transcription start sites, which were negatively correlated with gene expression differences, the widespread methylation changes from P12 to P35 (Fig. 1C) were not clearly correlated with expression changes (fig. S4, D and E). This suggests that, rather than regulating steady-state expression, postnatal epigenetic maturation in the ARH regulates gene expression potential, enabling adult-like responses to circulating hormones and nutrient signals.

### Postnatal epigenetic maturation is associated with the response to food restriction

**Fig. 3.**
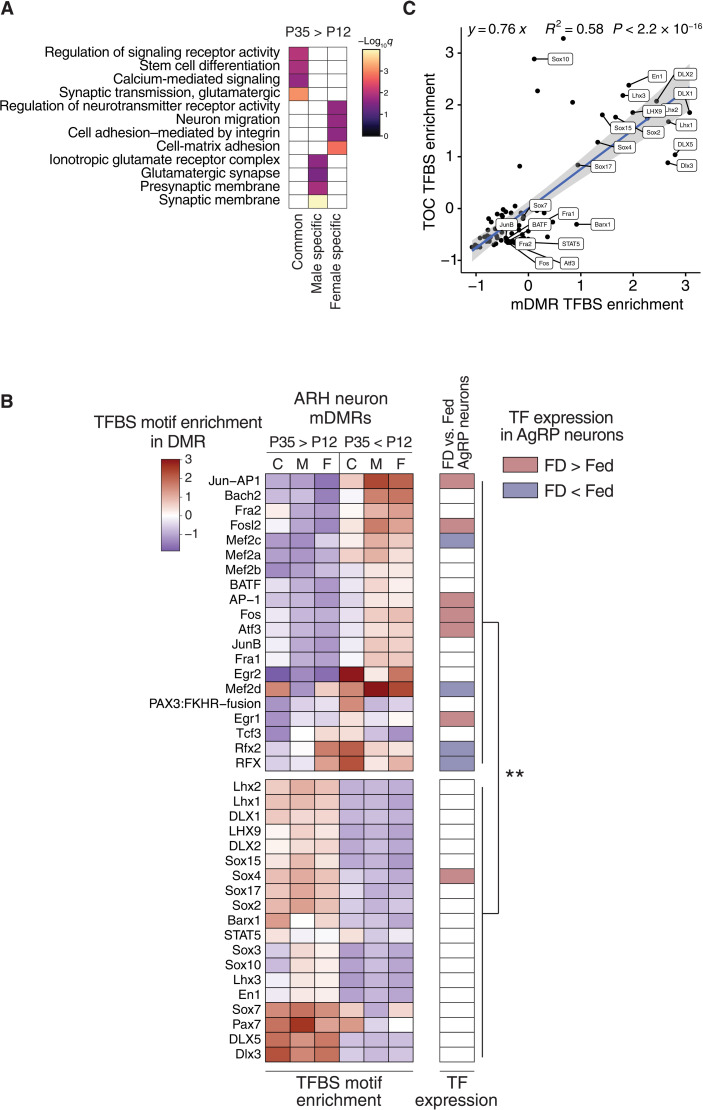
Postnatal epigenetic maturation in ARH neurons is associated with maturation of the response to dietary signals. (**A**) GO biological function analysis of genes associated with common and sex-specific P35 > P12 neuron mDMRs. (**B**) Heatmap illustrating scaled enrichment of TF binding site motifs among neuron mDMRs that increase (P35 > P12) or decrease (P35 < P12) with age (left). *K*-means clustering (*k* = 2) divides these into two groups (top and bottom). Food deprivation–induced differential expression of TFs in AgRP neurons (right) (*46*). TFs regulated by food deprivation (FD) are enriched among the motifs associated with P35 < P12 mDMRs (χ^2^ = 10.16, *P* < 0.01). (**C**) Scaled motif enrichment in male neuron P35 > P12 TOCs is highly correlated with that at mDMRs (*P* < 2.2 × 10^−16^).

To explore the function of postnatal epigenetic maturation in the ARH, we analyzed gene ontology (GO) terms associated with mDMRs (Fig. 3A and fig. S5A). In neurons, among mDMRs reflecting methylation increases (the majority; Fig. 1E), those common to both sexes were associated with stem cell differentiation, consistent with commitment to developmental fate (Fig. 3A). Those specific to males were associated with glutamatergic synapses (Fig. 3A), providing a potential explanation for the elevated excitatory tone in female versus male proopiomelanocortin (POMC) neurons (*35*, *36*).

Neuronal differentiation involves stage-specific methylation changes at enhancers and transcription factor (TF) binding sites, epigenetically “priming” cell type–specific patterns of methylation-sensitive enhancer activity and TF binding (*23*, *37*–*40*). Consistent with observations in the mouse cortex (*23*, *41*), both mDMRs and TOCs were enriched for various sets of brain enhancers (fig. S5D). We next asked whether neuronal mDMRs are enriched for TF binding motifs. mDMRs undergoing methylation increases were enriched for binding motifs of neurodevelopmental TFs in the *Lhx*, *Dlx*, and *Sox* families (Fig. 3B, bottom section) involved in hypothalamic development (*42*–*44*). Those that lost methylation, on the other hand, were enriched for binding motifs of key immediate early gene TFs that are markers for neuronal activity (Fig. 2B, top section), including AP-1, *Fos*, *Jun*, *Egr*, and *Atf* (*45*). Notably, half of these are transcriptionally regulated in AgRP neurons in response to food deprivation (Fig. 3B) (*46*) versus only one of the TFs associated with methylation gains (χ^2^ = 10.16, *P* < 0.01; Fig. 3B). TF chromatin immunoprecipitation sequencing (ChIP-seq) data from the brain (fig. S5B) corroborated the motif-based analysis. These data suggest that postnatal maturation of the transcriptional response to fasting (a key function of ARH neurons) involves targeted demethylation at binding sites for fasting-induced TFs. Compared to mDMRs, the TOCs identified by our read-level analysis showed markedly similar patterns of enrichment and depletion of TF binding motifs in both male (Fig. 3C) and female neurons (fig. S5C), supporting our interpretation that TOCs represent cell type–specific mDMRs. Although both mDMRs and TOCs are largely sex specific, associations with TF binding do not show substantial sex differences. The ability of ARH neurons to regulate energy balance in response to peripheral signals develops after the second postnatal week in rodents (*47*); together, therefore, our data suggest a key role for epigenetics in the genesis of energy balance regulation.

### Sex-specific epigenetic maturation occurs at human GWAS loci linked to BMI

A recent meta-analysis of GWAS for BMI comprising data on approximately 700,000 individuals found that BMI-associated variants are enriched among brain-associated and neurodevelopmental genes (*9*). We therefore wondered whether BMI-associated single-nucleotide variants (SNVs) are associated with ARH mDMRs. Because phenotype-associated genetic variants often affect cell type–specific genes or regulatory elements (*48*), we likewise tested for associations with TOCs. To confirm that it is reasonable to map mouse mDMRs and TOCs to the human genome, we evaluated the PhastCons score, a measure of evolutionary conservation, in the genomic regions we identified, finding that mDMRs and TOCs tend to exhibit higher conservation than neuron-versus-glia DMRs (table S5). This is particularly compelling given that neuron-versus-glia DMRs, which are established in the mouse hypothalamus during early postnatal life (*28*), are highly conserved between mouse and human (*32*, *49*), suggesting both that our findings in mice are relevant to humans and that genetic variation in these elements could be particularly deleterious to neurodevelopment.

**Fig. 4.**
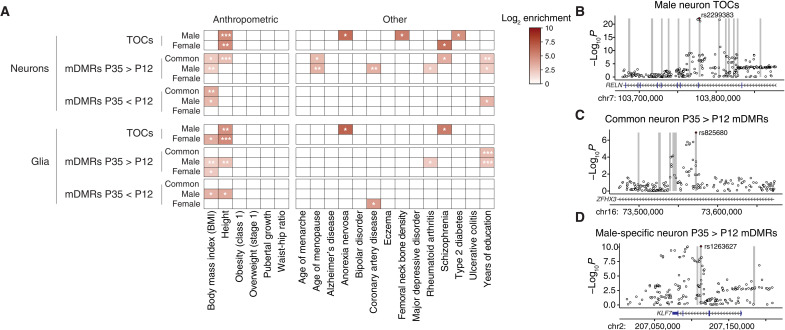
Regions of sex-specific epigenetic maturation in mouse ARH are enriched for heritability of BMI in humans. (**A**) Significant enrichment of BMI-associated GWAS SNVs in mDMRs and TOCs as computed by stratified LD score regression. Only enrichments remaining significant after Benjamini-Hochberg correction are shown. (**B** to **D**) Examples of overlaps between TOCs or mDMRs and NHGRI index SNVs associated with BMI. Human orthologs of mDMRs and TOCs (±1 kb) are indicated by vertical gray bars. Benjamini-Hochberg–adjusted *P* values for SNV-BMI associations obtained from (*9*). *P < 0.05, **P < 0.01, and ***P < 0.001.

To evaluate, we used stratified linkage disequilibrium score regression (*10*) to determine whether heritability for a range of traits is enriched in human orthologs of mDMRs and TOCs (table S6). To control for potential overlap with the 53 baseline epigenomic features from Finucane *et al.* (*10*), we first ran this analysis using just mDMR and TOC features (Fig. 4A). We then included the baseline features from Finucane *et al.* (*10*) (baseline-included model; fig. S6A). SNV heritability for BMI was enriched in male-specific and common mDMRs that gain methylation in postnatal ARH neurons (Fig. 4A), a finding that persisted in the baseline-included model (fig. S6A). Male-specific neuronal TOCs were strongly associated with heritability for height (Fig. 4A) and, in the baseline-included model, both height and BMI (fig. S6A). Concordance between the models indicates that variants within mDMR and TOC regions are associated with additional heritability beyond that explained by the baseline epigenomic features. We considered whether the frequent co-enrichment of both BMI and height may reflect that height is used in the calculation of BMI. Index SNVs driving associations of mDMRs and TOCs with height, however, are generally distinct from those driving associations with BMI (fig. S6, C and D). Female-specific TOCs in glia (but not neurons) were enriched for heritability of BMI in both models (Fig. 4A and fig. S6A) and, in the baseline-included model, for pubertal growth (fig. S6A), suggesting a previously unrecognized role for ARH glia in sex differences in somatic growth.

In addition to overlap with genetic variants associated with BMI, we also considered overlap with regions known to exhibit differential DNA methylation in the ARH in animal models of postnatal developmental programming (*50*–*55*). Notably, these regions are significantly enriched within neuron-versus-glia DMRs, as well as within male-specific neuron TOCs (fig. S6, E and F), suggesting that the genomic regions we have identified constitute promising targets for future epigenetic investigations into the developmental programming of energy balance.

## DISCUSSION

Our study, the first unbiased, genome-wide, cell type– and sex-specific analysis of epigenetic maturation in the mouse ARH, supports the hypothesis that the suckling period is a critical window for epigenetic developmental programming of energy balance regulation. We show that in the mouse ARH, neurons and glia undergo sex-specific epigenetic maturation during early postnatal life. These previously unknown sex differences echo well-known neuroanatomic and functional sexual dimorphism in the hypothalamus. Relative to males, female rodents have more axosomatic and fewer axodendritic synapses onto ARH neurons (*56*) and are more responsive to leptin (*57*), both of which are stable sexual dimorphisms established in early postnatal life. Females also have more POMC neurons, express higher levels of POMC, and have greater numbers of excitatory glutamatergic synapses onto POMC neurons (*36*). Although less studied in the context of energy balance, ARH glia are also sexually dimorphic. Compared to males, female rats have simpler astrocyte morphology in the ARH (*58*) and, in the hypothalamus, a reduced inflammatory response to high-fat diet (*59*). By studying the developmental dynamics of CpG methylation, we were able to show that epigenetic development of the ARH is advanced in females relative to males, expanding upon previous observations of sex differences in hypothalamic methylation (*31*, *60*).

**Fig. 5.**
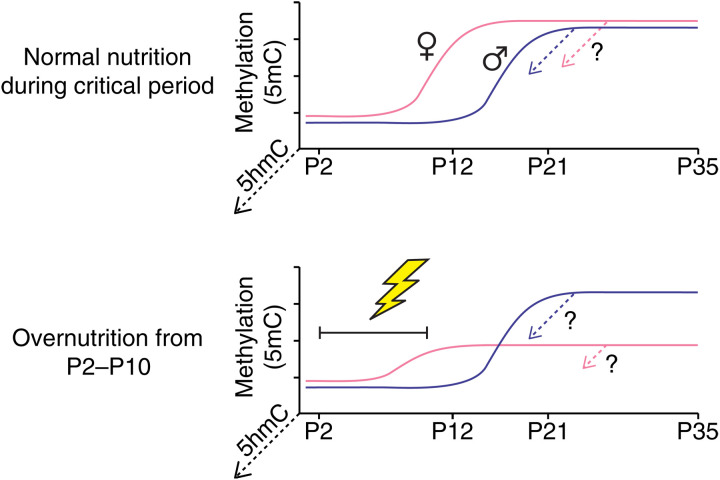
Conceptual model for how sex-specific epigenetic development in the ARH could underlie sex differences in susceptibility to developmental programming. Illustrations depict early postnatal methylation dynamics at a female-precocious neuronal mDMR (i.e., one at which methylation increases earlier in female than in male ARH neurons). Under normal postnatal nutrition (top), the same developmental outcome is achieved in both male and female ARH neurons (potential conversion of some nascent 5mC to 5hmC is illustrated by dotted arrows projecting out of the page). In this hypothetical example, overnutrition from P2 to P10 (bottom) impairs de novo methylation only in females, because this epoch overlaps the critical window for ARH neuronal epigenetic maturation at this female-precocious locus.

Critical windows for environmental influences on epigenetic regulation coincide with ontogenic periods when DNA methylation is undergoing developmental establishment or maturation (*24*, *25*). Disruption of these maturational processes offers a potential explanation for the persistence and sex specificity of developmental programming by postnatal nutrition (Fig. 5) (*26*). Our results also provide mechanistic insight into the function of postnatal epigenetic development in the ARH. Binding sites for TFs in the *Lhx*, *Dlx*, and *Sox* families gain methylation in ARH neurons postnatally (Fig. 3B). These TFs are associated with neuronal differentiation, migration, and neurite outgrowth (*61*–*63*) and specify subtypes of ARH neurons (*42*). At the same time, binding sites for TFs such as *Fos*, *Egr1*, *Jun*, and others lose methylation in ARH neurons. These activity-dependent immediate early genes, many of which are regulated by fasting in AgRP neurons in the ARH (*46*), organize rapid transcriptional responses to stimulus-induced neural activity (*64*). Because TF binding is frequently methylation sensitive (*38*), together, these observations indicate that postnatal epigenetic maturation in ARH neurons reprograms transcriptional regulatory networks, silencing those previously required for neural growth and differentiation and derepressing those responsive to acute energy balance states. This maturation of gene expression potential may explain why mDMR methylation changes were generally not associated with gene expression. Elucidating such associations may require perturbations, such as fasting, designed to elicit hormonal and nutritional signaling from the periphery.

GWAS results strongly indicate a neurodevelopmental etiology for obesity (*8*, *9*). Our data indicating that sex- and cell type–specific epigenetic development in the ARH occurs in genomic regions associated with heritability of human BMI provide further evidence, ultimately suggesting that adult obesity risk is determined in part by epigenetic development in the ARH. This is consistent with the observed enrichment of BMI heritability within human fetal brain H3K4me3 (*10*). Our data also corroborate earlier studies indicating that neuroepigenetic development and regulatory features are highly conserved from mouse to human (*32*, *65*), indicating that the mouse provides an apt model for human epigenetic development in the ARH. Many targets for postnatal epigenetic development, including neuron mDMRs and TOCs, remain significantly enriched for BMI heritability even when highly enriched baseline factors such as conservation and non–tissue-specific epigenetic features are included in the model. This indicates that our findings are not an artifact of heightened conservation in regulatory regions.

We believe that public health interventions to curb the worldwide obesity epidemic would benefit by considering obesity as a neurodevelopmental disorder. For decades, investigations into the developmental origins of health and disease have documented the outsized effects of environmental exposures during early life (*3*, *4*, *66*). The more recent BMI GWAS data (*8*, *9*) provide independent corroboration that human obesity is strongly determined during prenatal and early postnatal development. Our results provide the novel insight that developmental epigenetics are likely involved in both early environmental and genetic influences on obesity risk, suggesting that improved understanding of these cell type–specific developmental processes could offer insights into effective primary prevention of obesity. Recently, however, such studies have not been prioritized. Instead, research into the epigenetics of obesity has increasingly focused on studies of peripheral tissues in humans. For example, a genome-scale study screening for associations between peripheral blood DNA methylation and BMI concluded that interindividual epigenetic differences are a consequence rather than a cause of obesity (*67*). While studies of this sort are valuable in elucidating biomarkers and epigenetic sequelae of obesity, they highlight the importance of studying the brain to understand the etiology of obesity.

Building a mechanistic understanding of the neuroepigenetic basis of obesity will require initial studies in animal models to identify the ontogenic periods, brain regions, and cell types involved. The technologies needed for this sort of genome-wide, brain region– and cell type–specific study of epigenetic development are only recently available. The best previous work surveying postnatal epigenetic development in the mouse brain (*23*) used FANS, WGBS, and RNA-seq to compare neurons and glia from the cerebral cortex of adult versus fetal mice. That study, however, was neither designed to resolve sex differences in early postnatal epigenetic development nor targeted to a brain region central to energy balance regulation. Neuronal DNA methylation differs widely between brain regions (*49*), highlighting the importance of studying regions appropriate to the phenotype of interest. Studying sex differences is similarly important, as foundational studies focused on the preoptic area of the hypothalamus found that gonadal steroids play a role in establishing sex differences in DNA methylation (*31*, *60*), raising the possibility that similar mechanisms are at work elsewhere in the brain. A recent study of pubertal epigenetic development in the female mouse ARH attests to the importance of DNA methylation in mediating postnatal hypothalamic development, again pointing to a role for epigenetics in mediating sex-specific neurodevelopmental processes, but lacks genome-wide coverage and cell type–specific resolution (*30*). Hence, while there is longstanding interest in the idea that the development of energy balance regulation involves epigenetic mechanisms in the ARH, the lack of a “roadmap” has forced investigators to generally focus on candidate genes such as *Pomc* (*68*) and *Agrp* (*53*). These previously studied regions are enriched within our neuron-versus-glia DMRs and male neuron TOCs, suggesting that our unbiased and sex-specific genome-wide data on neuron- and glia-specific epigenetic development in the ARH provide valuable targets for future studies.

Our study is not without limitation. Although we did study neurons and glia separately, these classes are themselves heterogeneous (*34*). Interpreting developmental dynamics in WGBS data is complicated by the possibility that proportions of different cell types may change between two time points. This can make it difficult to distinguish methylation changes occurring within cells from those reflecting shifts in the proportional representation of different cell types. In this regard, it is advantageous that hypothalamic neurons are postmitotic by P12 (*69*); hypothalamic glia, however, are still a dynamic population at this time, complicating interpretation. Read-level analysis of our WGBS data provided evidence of methylation changes occurring within only specific neuronal subclasses. With just two time points, however, we had limited power to deconvolute methylation dynamics. Future studies obtaining WGBS data on many more time points will enable the application of latent-state computational models (*70*) to identify subclasses of ARH neurons following distinct developmental trajectories.

As noted above, conventional bisulfite sequencing cannot distinguish between cytosine methylation and hydroxymethylation. Accordingly, steady-state methylation at any given region could be a combination of both methylation and hydroxymethylation (*27*). Hydroxymethylation (5hmC) is an intermediate in the Tet-mediated demethylation pathway, but accumulating evidence indicates that it also acts as an epigenetic mark (*71*, *72*). Compared to 5mC, 5hmC is relatively enriched in cis-regulatory elements and may play a role in regulating neural cell type–specific gene expression (*27*). Previous studies in the mediobasal hypothalamus show that *Tet* expression and enzymatic activity decline substantially by P25 in mice (*60*), suggesting limited formation of 5hmC in the ARH within the ontogenic window we studied. However, we do observe evidence of modest demethylation from P12 to P35 (Fig. 1, E and F), and this could be an underestimate owing to the inability to distinguish 5mC from 5hmC. In contrast, because unmethylated cytosine is not a substrate for hydroxylation by Tet enzymes, methylation increases from P12 to P35 (the vast majority of features we identified; Fig. 1, C to F) must reflect actual gains in 5mC; whether these stabilize as 5mC or 5hmC, however, remains to be determined (Fig. 5). Future studies incorporating measures of 5hmC and including additional time points flanking ours would be valuable to address this. As another potential limitation, our study focused on the C57BL/6 mouse, the most widely used laboratory strain. While approximately half of mDMRs exist in genomic regions that are conserved between mice and humans (table S5), and neuron-versus-glia differential methylation is conserved between mice and humans (*32*), methylation differences do occur between strains (*73*), so epigenetic maturation may also differ. Given the wide range of variation in energy balance phenotype between strains (*74*), a comparative approach would be informative. Last, sex differences in neuroepigenetics are evident at birth, around the time of the gonadal hormone surge (*60*), and continue to evolve into adulthood (*75*), necessitating a more detailed ontogeny to fully understand observations such as precocious epigenetic development in females, which could help explain differential vulnerability to obesity (*76*), and developmental programming (*26*, *77*, *78*) in females, as well as the sex-specific genetic architecture of disease risk (*79*, *80*) in general.

Our data provide a framework and novel set of candidate regions for both investigating the epigenetic basis of developmental programming and translating findings from rodent models to humans. The overlap with human BMI GWAS SNVs points to the need to determine when analogous developmental neuroepigenetic processes occur in humans, which would point to the optimal time for intervention. Of major concern in humans, long-term effects of maternal obesity during offspring fetal development (*81*) may lead to transgenerational amplification of obesity (*15*). Because many neurodevelopmental processes that occur postnatally in mice occur during late fetal development in humans, it is possible that the ARH neuroepigenetic maturation we describe here occurs in utero in humans.

In summary, our study, built on the premise that obesity is a neurodevelopmental disorder, highlights the power of a developmental neuroepigenetic perspective to contextualize GWAS results and identify ontogenic processes vulnerable to developmental programming. As obesity remains a major public health challenge, we hope that our work will invigorate efforts to understand developmental determinants of obesity risk.

## MATERIALS AND METHODS

### Animals

This study was approved by the Institutional Animal Care and Use Committee of Baylor College of Medicine and Vanderbilt University, and animals were maintained in accordance with federal guidelines. Female C57BL6/J mice aged 2 to 4 months were mated and singly housed with ad libitum access to food and water for the duration of pregnancy and lactation. Litters of six to nine pups were used for this study. ARH was microdissected as described in (*82*) from brains collected at P12 and P35 from randomly selected male and female mice (*n* = 1 per sex per litter). ARH microdissections were pooled to generate five independent tissue pools with *n* = 4 mice each per age and sex, yielding a total of 20 tissue pools. Tissue pools were flash-frozen on dry ice and stored at −80°C.

### Tissue preparation

NeuN immunolabeling and FANS were carried out as previously described (*82*). Pooled ARH microdissections were dissociated by Dounce homogenization. Nuclei were purified by ultracentrifugation on a 1.8 M sucrose column (100,000 rcf). Pelleted nuclei were collected and stained using rabbit anti-NeuN (1:4000; Millipore, ABN78) followed by Alexa Fluor 488–conjugated goat anti-rabbit immunoglobulin G (1:2000; Thermo Fisher Scientific, A-11008) and the nucleic acid dye TO-PRO-3 as a counterstain (1:1000; Thermo Fisher Scientific). Sorting was performed on the Sony SH800 Cell Sorter (Sony Biotechnology). FSC (Forward scatter) and SSC (Side scatter) gates were set to exclude debris and non-nuclear material, and TO-PRO-3 nuclear gating was used to collect single nuclei only (fig. S1A). Sorted nuclei were pelleted and frozen on dry ice and stored at −80°C. DNA was extracted from sorted nuclei using the AllPrep DNA/RNA Micro Kit (QIAGEN) according to the manufacturer’s directions. RNA was collected from flow-through after DNA isolation using TRIzol LS according to the manufacturer’s instructions (Thermo Fisher Scientific). DNA was eluted from columns using two rounds of 50 μl of nuclease-free H_2_O (pH 8.0), dried in SpeedVac (Eppendorf), and then resuspended in 12 μl of TE buffer (pH 8.0). DNA and RNA were quantitated by PicoGreen and NanoDrop, respectively.

### RNA sequencing

Libraries for RNA-seq were prepared using the SMART-Seq v4 Ultra Low Input kit (Takara Clontech) according to the manufacturer’s instructions (*82*). RNA was incubated with lysis buffer for 5 min. First-strand complementary DNA (cDNA) synthesis was performed using the included 3-SMART-seq CDS primer II and V4 oligonucleotide. cDNA was amplified using polymerase chain reaction (PCR) Primer II A and subsequently purified using AMPure XP beads (Beckman). Illumina libraries were prepared using a Nextera XT DNA library preparation kit (Illumina) and sequenced on the Illumina HiSeq 2500 platform generating 100-bp paired-end reads.

### Whole-genome bisulfite sequencing

Tagmentation-based WGBS library preparation was performed as previously described (*82*, *83*) using 50 to 100 ng of mouse genomic DNA. After adapter preannealing and transpososome assembly, DNA was tagmented by adding the assembled transpososome and incubating at 55°C for 8 min. Following purification using SPRI beads, oligonucleotide replacement and gap repair were performed using Ampligase and T4 DNA Polymerase. The product was purified using SRPI beads and followed by bisulfite treatment using the EZ DNA Methylation Gold Kit (Zymo Research). Libraries were generated by PCR amplification using KAPA 2G robust hotstart ready mix. After purification using SPRI beads, libraries were diluted and sequenced on a NovaSeq 6000 platform generating 150-bp paired-end reads.

### RNA-seq analysis

Read quality was analyzed before and after trimming using Fastqc (v0.11.5). RNA-seq reads were aligned to the mouse reference genome GRCm38v23 using STAR v2.7.9a (*84*). Reference sequence and annotations were downloaded from GENCODE portal. Genome was indexed using STAR by setting --runMode to genomeGenerat. Gene expression values from each sample were quantified as the number of reads mapped (to a specific gene) by setting --quantMode to GeneCounts. --outFilterScoreMinOverLread and --outFilterMatchNminOverLread were set to 0.3. Sample numbers used in the final analysis are glia_f_12 = 5, glia_m_12 = 5, glia_f_35 = 5, glia_m_35 = 5, neuron_f_12 = 5, neuron_m_12 = 5, neuron_f_35 = 5, and neuron_m_35 = 4. Normalization and differential expression testing were performed using DESeq2 (v1.28.1) using cell type, age, and sex as factors (*85*). Pairwise comparisons were made using the apeglm package for log_2_ fold change shrinkage (*86*). Significant DEGs were defined as those with abs(log_2_FoldChange) > 1 and Benjamini-Hochberg–adjusted *P* value of <0.05.

### WGBS analysis

FASTQ files were quality-trimmed using TrimGalore (v0.4.4) with a minimum Phred score of 20 and a minimum posttrimming read length of 50. Read quality was analyzed before and after trimming using Fastqc (v0.11.5). Libraries were deduplicated using Picard (v2.10.10). Trimmed reads were aligned to the mouse genome (mm10) with Bismark (v0.18.1) using the default settings (*87*). Sample numbers used in the final analysis are glia_f_12 = 5, glia_m_12 = 4, glia_f_35 = 4, glia_m_35 = 4, neuron_f_12 = 5, neuron_m_12 = 4, neuron_f_35 = 5, and neuron_m_35 = 3. Autosomal CG, CHG, and CHH methylation calls were gathered from Bismark output and used to assess broad developmental changes in cytosine methylation using unpaired two-sided *t* tests. Differentially methylated CpG loci (DMLs) were called using DSS (*88*) first using the general experimental design with cell type, age, and sex as factors to determine the relative contribution of each to the per-CpG methylation state, followed by pairwise comparisons between P12 and P35 libraries separated by cell type and sex. Only CpGs with at least five reads per library were considered. Across all autosomal CpGs, the average depth for individual libraries was 16.6× (± 5.2) and for combined library groups was 77.3× (± 13.8). Libraries with less than 10× average depth across all autosomal CpGs were excluded from analysis. DMRs were called from pairwise DML comparisons using DSS with an FDR threshold of 0.05 and a minimum absolute differential methylation cutoff of 5%. DMRs were annotated to UCSC gene features with promoter and 3′ regions defined as regions respectively flanking the TSS (Transcription Start Site) and TES (Transcription End Site) by 3 kb. The upstream and downstream annotations correspond to the 7 kb up- or downstream of the 5′ or 3′ flank of the promoter and 3′ annotation, respectively. Unless otherwise noted, all methylation analyses are conducted solely on autosomes.

### Initial clustering of WGBS and RNA-seq data

CpGs meeting coverage criteria were grouped into 1-kb genomic bins. Average autosomal bin-level methylation was calculated for each sample. Before clustering, the first principal component was removed from the dataset using the removePrincipalComponents function in the WGCNA (v.1.70.3) package. Surrogate variable analysis was conducted using the R package sva (v.3.36.0) to remove bins with >0.25 probability of being associated with one or more latent variables (pprob.gam) and more than >0.75 probability of being associated with one or more of cell type, age, or sex (pprob.b), yielding 706,217 of 1,405,375 1-kb bins used for clustering. The R package umap (v.0.2.7.0) was used to cluster scaled methylation values with the following settings: min_dist = 0.25, spread = 0.5, and n_components = 2. This analysis was repeated on RNA-seq data with pprob.gam < 0.5 and pprob.b > 0.5, yielding 18,260 of 26,578 transcripts used for umap clustering.

### Comparison with cortical DMRs

CpG methylation calls were obtained from mouse frontal cortex neurons and glia (*23*). This dataset was first lifted over to mm10 and then processed through our DSS pipeline using the same depth, significance, and differential methylation cutoff criteria as our ARH neurons and glia to obtain neuron-versus-glia DMRs in the cortex. DMR sets were first reduced to include only those overlapping regions meeting read depth criteria in both ARH and cortex datasets. The statistical significance of overlap between cortical DMRs and our ARH DMRs was calculated using R package LOLA (v.1.18.1) (*89*) with all CpG-bearing 100-bp genomic bins meeting coverage criteria in both ARH and cortex as the universe region set. Mouse cortical neuron-versus-glia DMRs and nondifferentially methylated control regions were validated by bisulfite pyrosequencing in (*32*). These genomic regions were lifted over to mm10 and compared with our ARH neuron-versus-glia DMRs using a chi-square test of independence.

### Correlation of DNA methylation and gene expression

Per-sample methylation values within DMR loci were correlated with per-sample gene expression values in TSS-flanking bins using Spearman correlation. Scrambled correlations were generated as a control using random DMR-gene pairings. To illustrate the relationship between methylation and expression in promoter regions, several representative correlations were chosen from promoter DMRs in the neuron-versus-glia and common neuron mDMR context. Only sample pools for which both RNA-seq and WGBS data were available were used in this analysis (table S1).

### CluBCpG analysis

Read-level analysis using the CluBCpG package was preceded by random forest imputation of missing methylation calls from reads mapped to nonoverlapping 100-bp bins genome wide [Precise Read-Level Imputation of Methylation (PReLIM)] (*33*). This yielded a total of 3,793,207 covered autosomal bins in neurons and 3,793,867 in glia. Sample libraries with the same cell type, age, and sex were combined, yielding merged libraries for comparison using CluBCpG. The original implementation of CluBCpG was designed to compare libraries with roughly equivalent read depths and had no framework for making statistical comparisons in epiallele frequency. To account for this, we developed a down-sampling approach to read depth normalization. For each bin, we identified the merged library with the lowest read depth and set that value as the target. Reads from merged libraries were resampled 100 times per bin, drawing a number of reads equal to that bin’s target. For each bin, we calculated the proportion of the down-sampled reads made up of each epiallele and compared this value between libraries. Within each bin, epialleles that were significantly more frequent in one library were defined as enriched. The statistical significance of this enrichment value was determined using two-sided Fisher’s exact test with a significance threshold of *P* < 0.05 for each of the 100 draws, and read clusters that were significant on >95% of draws were defined as significantly enriched in one library relative to the other. This process was conducted separately in neurons and glia, yielding an average of 54.01 informative reads per bin in neurons and 54.41 in glia. TOCs were defined as bins containing a fully methylated read cluster significantly enriched at P35 relative to P12. TOCs were generated in both sexes, and overlapping bins were excluded to yield sex-specific TOCs.

### Gene enrichment analysis

GO analyses were conducted using the R package clusterProfiler (v.3.16.1) (*90*). For RNA-seq analysis, significant DEGs were used as input, while for DMR analysis, genes annotated to promoter DMRs were used. Figures were generated using the top 4 most significantly enriched GO Biological Process terms.

### Genomic overlap and enrichment analysis

Analysis of DMR and TOC enrichment in genomic annotations was conducted using the R package LOLA (v.1.18.1) (*89*) with all covered, CpG-bearing 100-bp genomic bins as the universe region set. Annotations for ChIP-seq were obtained from the ChIP-Atlas (*91*) neural peak set on 11 November 2020 using a significance threshold of *q* < 1 × 10^−10^. Annotations for brain enhancers were derived from EnhancerDB (obtained on 29 March 2022) and lifted over to mm10 as needed (*92*).

### TF binding site motif enrichment analysis

We used HOMER (v.4.11.1) (*93*) to identify enriched motifs from the JASPAR CORE 2018 (*94*) database within our epigenetic maturation features (*93*). We ran the findMotifsGenome.pl function with the following flags: -size given -cpg -nomotif to generate CpG-matched background sets with the same size distribution as our methylation features. Enrichment was calculated as the ratio of motif matches in the target sequences to the number of matches in the background sequences. To compensate for the different sizes of methylation feature sets, enrichment values were scaled within each comparison for plotting. Heatmaps were plotted using the top 12 motifs by Benjamini-Hochberg–adjusted *P* value for each methylation feature and split by *k*-means clustering (*k* = 2). The proportion of fasting-regulated genes in each *k*-means cluster was tested using a chi-square test of independence.

### Enrichment of heritability in epigenetic maturation features

DMRs and TOCs were lifted over to hg19 using the UCSC liftOver software with a minimum of 50% matching bases, yielding an average of 54.62% of DMRs and TOCs successfully aligned to the human genome. We conducted stratified LD (linkage disequilibrium) score regression using the LDSC software (v.1.0.1) (https://github.com/bulik/ldsc) (*48*) to determine the enrichment of trait-associated heritability within our DMRs and TOCs. LD scores from a European population were downloaded from phase 3 of the 1000 Genomes project (https://data.broadinstitute.org/alkesgroup/LDSCORE/1000G_Phase3_plinkfiles.tgz). LD score regression was performed on HapMap3 single-nucleotide polymorphisms (SNPs) (https://data.broadinstitute.org/alkesgroup/LDSCORE/weights_hm3_no_hla.tgz) that are in the 1000 Genomes set excluding the major histocompatibility complex (MHC) region on chr6. We ran LDSC on all of our neuron and glia mDMRs and TOCs with 1-kb flanking regions, with and without the 53 “baseline” genomic features described in (*10*). Sources for summary GWAS statistics for traits are given in table S6. Enrichment scores and per-trait Benjamini-Hochberg–adjusted *P* values are reported for significantly enriched feature-trait combinations.

### Overlap of BMI and height SNPs

NHGRI (National Human Genome Research Institute) index SNPs for BMI and height were downloaded from the NHGRI database (27 October 2021). Expected overlap was defined as the proportion of overlapping BMI and height SNPs across the entire genome. Observed feature-associated overlap was computed in the same way, limited to SNPs overlapping mDMR and TOC features. Odds ratio was calculated as the ratio of observed, feature-associated overlap to expected genome-wide overlap. Two-sided Fisher’s exact test was used to test the hypothesis that mDMR and TOC features are enriched for SNPs that annotate to both BMI and height.

### Comparison with published developmental programming DMRs

The following search terms were entered into PubMed and Google Scholar (28 July 2020) to locate articles describing changes in DNA methylation in the ARH in response to models of nutritional developmental programming: (developmental programming, early-life, gestational diabetes, high-fat diet, high-growth diet, litter size, maternal diabetes, maternal obesity, maternal overnutrition, obesity, perinatal, postnatal, prenatal) + (CpG methylation, DNA methylation, epigenetic) + (arcuate nucleus, hypothalamus, mediobasal hypothalamus). Articles focusing on DNA methylation in the ARH in response to models of early-life programming were selected. Where possible, genomic ranges of regions showing significant differential methylation were extracted, lifted over to mm10 as needed, and expanded to a minimum size of 2 kb. To compute enrichment, null distributions were generated by creating 100 control sets for each type of DMR and TOC. Control sets were generated by randomly selecting, for each DMR/TOC, a genomic region of identical size and similar CpG density (±10%). Enrichment was calculated by comparing the overlap between published DMRs and ARH DMRs/TOCs relative to the mean overlap between published DMRs and the corresponding control sets. Statistical significance was calculated using a one-proportion *Z* test. Published DMRs included in this analysis are given in table S7.

### Software

All statistical analyses were performed in R (4.0.x). In addition to those packages listed above, we also used the following packages to prepare results and figures: Bsseq (v.1.24.4), data.table (v.1.14.0), dplyr (v.1.0.7), forcats (v.0.5.1), fuzzyjoin (v.0.1.6), genomicRanges (v.1.40.0), ggplot2 (v.3.3.5), ggpubr (v.0.4.0), pheatmap (v.1.0.12), and corpora (v.0.5).
