# Supplementary material for: Sex-specific epigenetic development in the mouse hypothalamic arcuate nucleus pinpoints human genomic regions associated with body mass index
Source: Sci Adv. 2022 Sep 28;8(39):eabo3991. doi: 10.1126/sciadv.abo3991 (PMC9519050; doi:10.1126/sciadv.abo3991)
Supplement: Supplementary file 1 — Figs. S1 to S6 [file sciadv.abo3991_sm.pdf]

Science Advances
AAAS

Supplementary Materials for

Sex-specific epigenetic development in the mouse hypothalamic arcuate nucleus pinpoints human genomic regions associated with body mass index

Harry MacKay *et al.*

Corresponding author: Robert A. Waterland, waterland@bcm.edu

Sci. Adv. **8**, eabo3991 (2022)
DOI: 10.1126/sciadv.abo3991

The PDF file includes:

Figs. S1 to S6
Legends for tables S1 to S7

Other Supplementary Material for this manuscript includes the following:

Tables S1 to S7

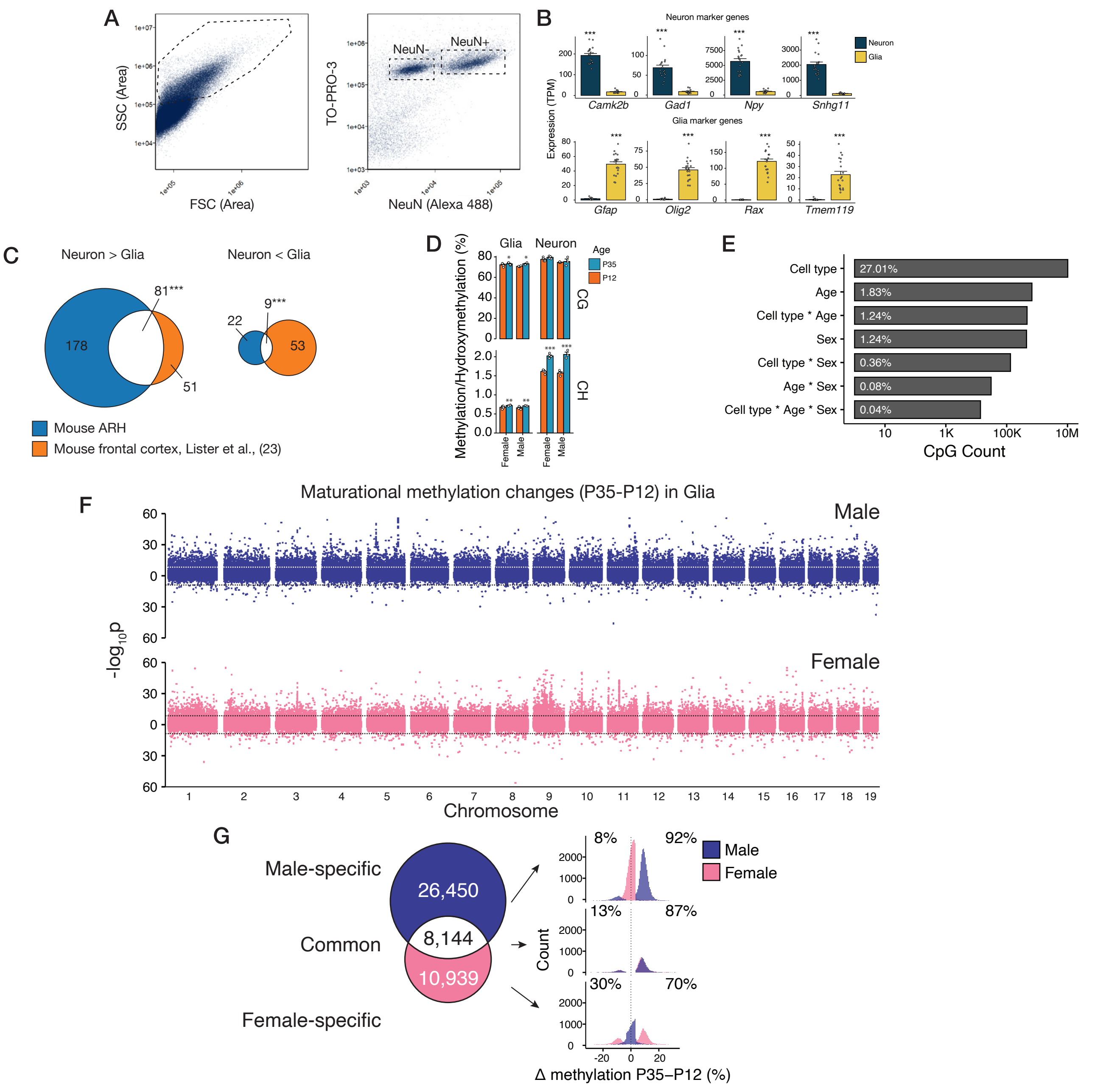

Figure S1 | Additional information associated with main Figure 1. A, Representative scatterplots of FANS sorting NeuN- and NeuN+ ARH nuclei. Dotted lines indicate gating strategy for FSC and SSC (left) and NeuN and TO-PRO-3 labeling (right). **B,** Mean expression (TPM) of cell type marker genes in isolated ARH neuronal and glial fractions validates effective sorting. *** $p<0.001$ neuron vs. glia differential expression. Error bars represent SEM. **C,** Venn diagram illustrating overlaps between the mouse neuron vs. glia DMRs we identified in ARH and those previously detected in frontal cortex. Genomic extent of each sector (Mb) is provided and statistical significance of overlaps indicated (*** $p<0.001$). **D,** Mean autosomal methylation/hydroxymethylation in CG and CH contexts in sample groups studied. * $p<0.05$, ** $p<0.01$, *** $p<0.001$ P12 vs. P35 methylation. Error bars represent SEM. **E,** Numbers of autosomal CpGs showing significant ($p<0.05$) effects of cell type, age, sex, or interactive effects. Percentages of all CpGs assayed are shown within bars. **F,** Miami plot showing significance of P35-P12 differential methylation, at the CpG level, in male and female glia. Points plotted above/below the origin represent CpGs that gained/lost methylation from P35 to P12, respectively. Y axis represents raw p-values. Genome-wide significance threshold for CpGs (horizontal dotted lines) = $p<2.45 \times 10^{-9}$. **G,** Venn diagram illustrating the overlap of mDMRs identified in male and female ARH glia. Histograms (right) show the magnitudes and directions of these maturational methylation changes. Median differential methylation for glial mDMRs ranges from 12-15%.

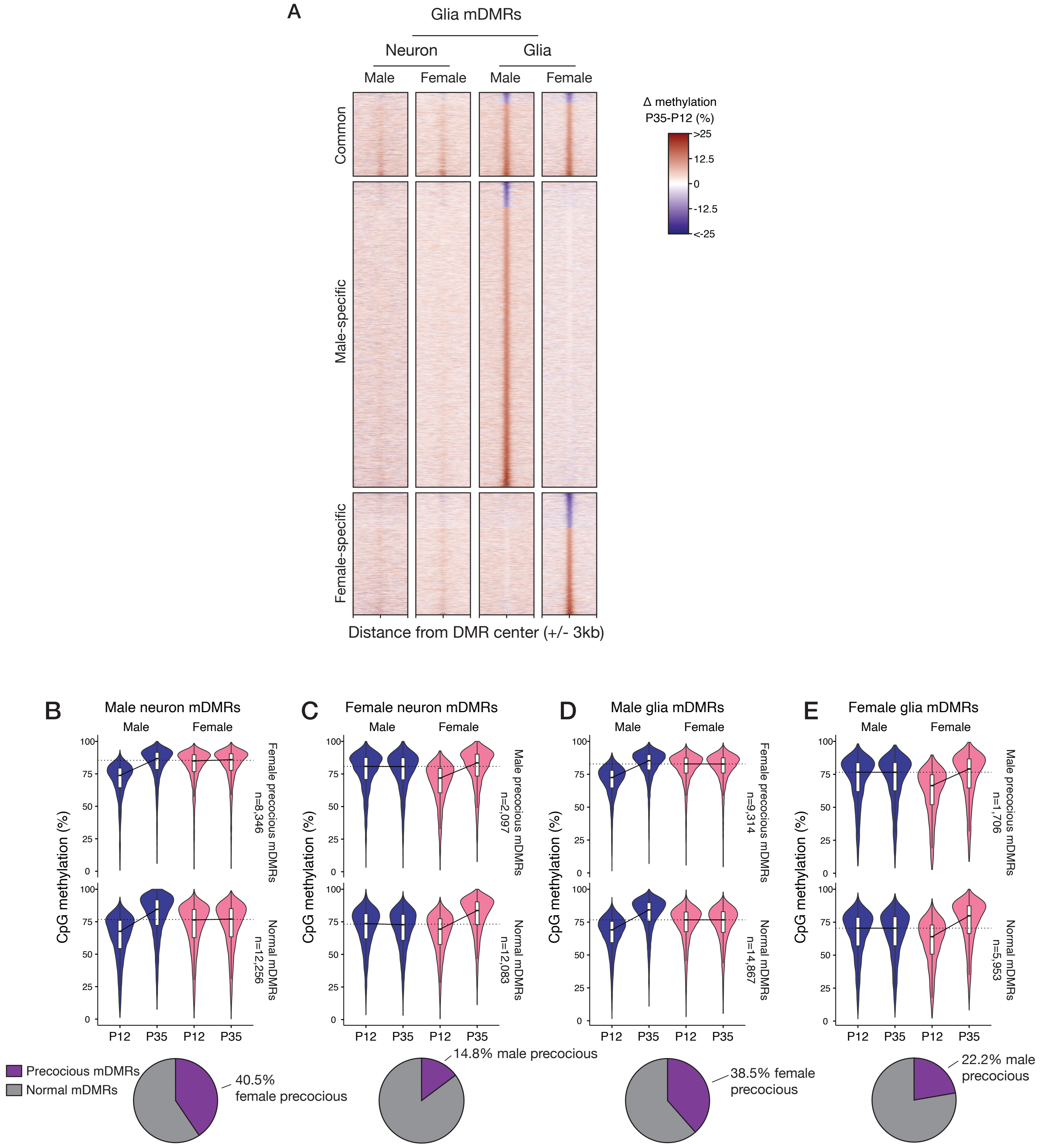

Figure S2 | Sex- and cell-type specificity of mDMRs. A, Differential methylation heatmaps centered on common and sex-specific mDMRs in ARH glia; most do not show maturational methylation changes in ARH neurons. **B-E,** Violin plots (top) summarize methylation dynamics at precocious and other (normal) mDMRs in neurons and glia. Boxplots overlaid represent median (+/- interquartile range) methylation. Average methylation levels at sex-specific P35>P12 mDMRs are plotted for males and females at each age. Dotted lines indicate median methylation in same cell type in other sex. Pie charts (bottom) show proportion of P35>P12 mDMRs that are precocious in each context.

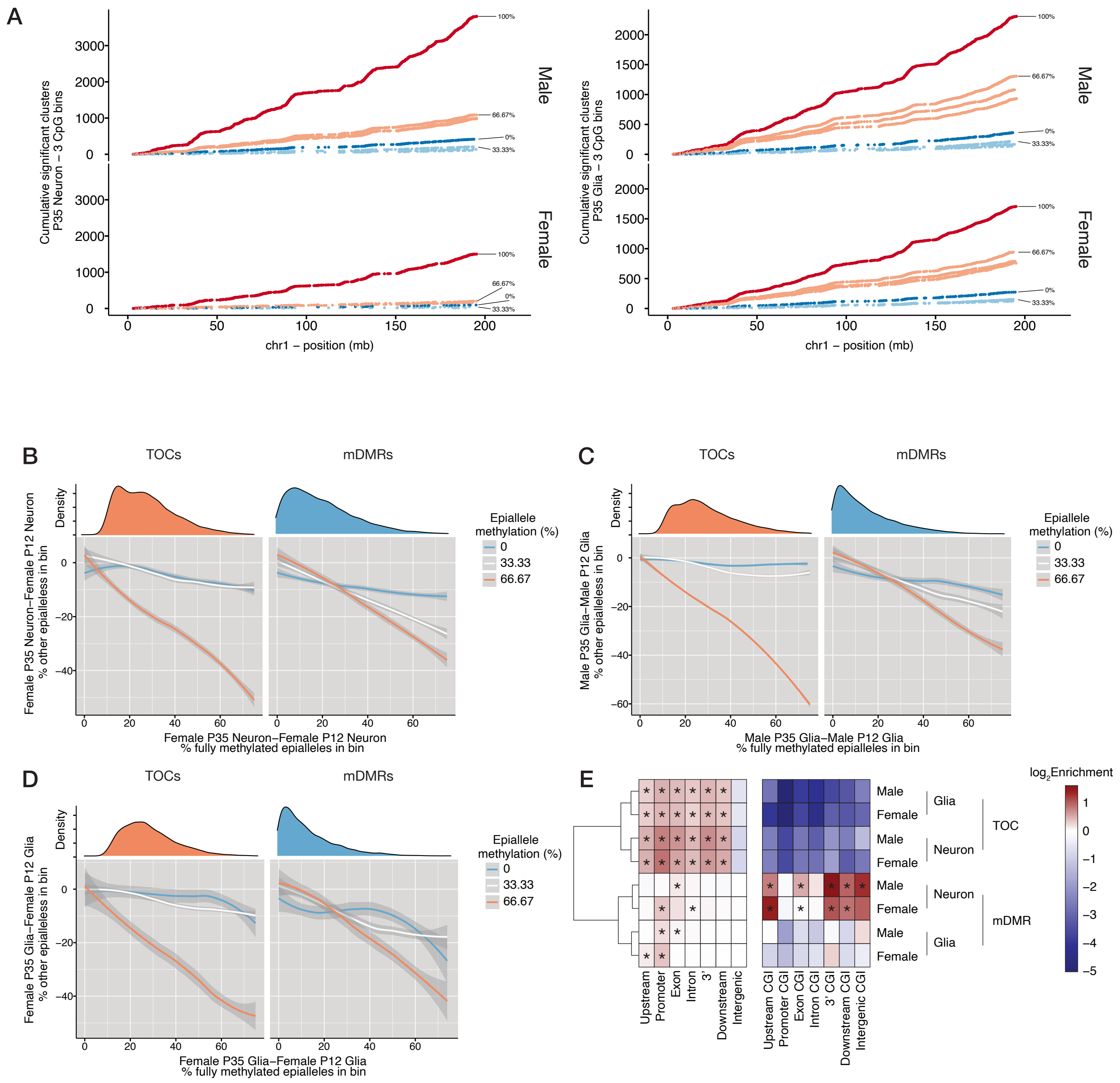

Figure S3 | Topped-Off Clusters (TOCs) are a feature of epigenetic maturation in neurons and glia. A, Cumulative frequency along chromosome 1 of epiallele methylation states enriched in P35 relative to P12 in neurons and glia. (Fig. 2B shows similar plots for chromosome 19.) Data are shown for 3-CpG genomic bins by bin average methylation (0%: 000; 33.3%: 100, 010, or 001; 66.7%: 101, 110, or 011; 100%: 111; unmethylated/methylated CpGs represented by 0/1). In both cell types, the dominant feature is enrichment of new 100% methylated clusters. Similar patterns are observed in all autosomes. **B-D,** For such regions, proportional losses of partially methylated clusters (y axis) are plotted vs. corresponding gains of fully methylated clusters at P35 relative to P12. The steep orange line indicates that most increases in fully methylated clusters arise from 2/3 methylated clusters (i.e. just one additional CpG site methylated); we call these 'topped off clusters' (TOCs). By contrast, at mDMRs (right), gains in fully methylated clusters appear to arise from both 1/3 and 2/3 methylated clusters. Shown are data on female neurons (**B**), male glia (**C**), and female glia (**D**). Density distributions (top) show that most TOCs involve fewer than 40% of the reads in each bin. **E,** Heatmap depicting opposing enrichments of P35 > P12 TOCs and mDMRs, respectively, in genic and CpG island (CGI) contexts. *Bonferroni-adjusted p-value < 0.05.

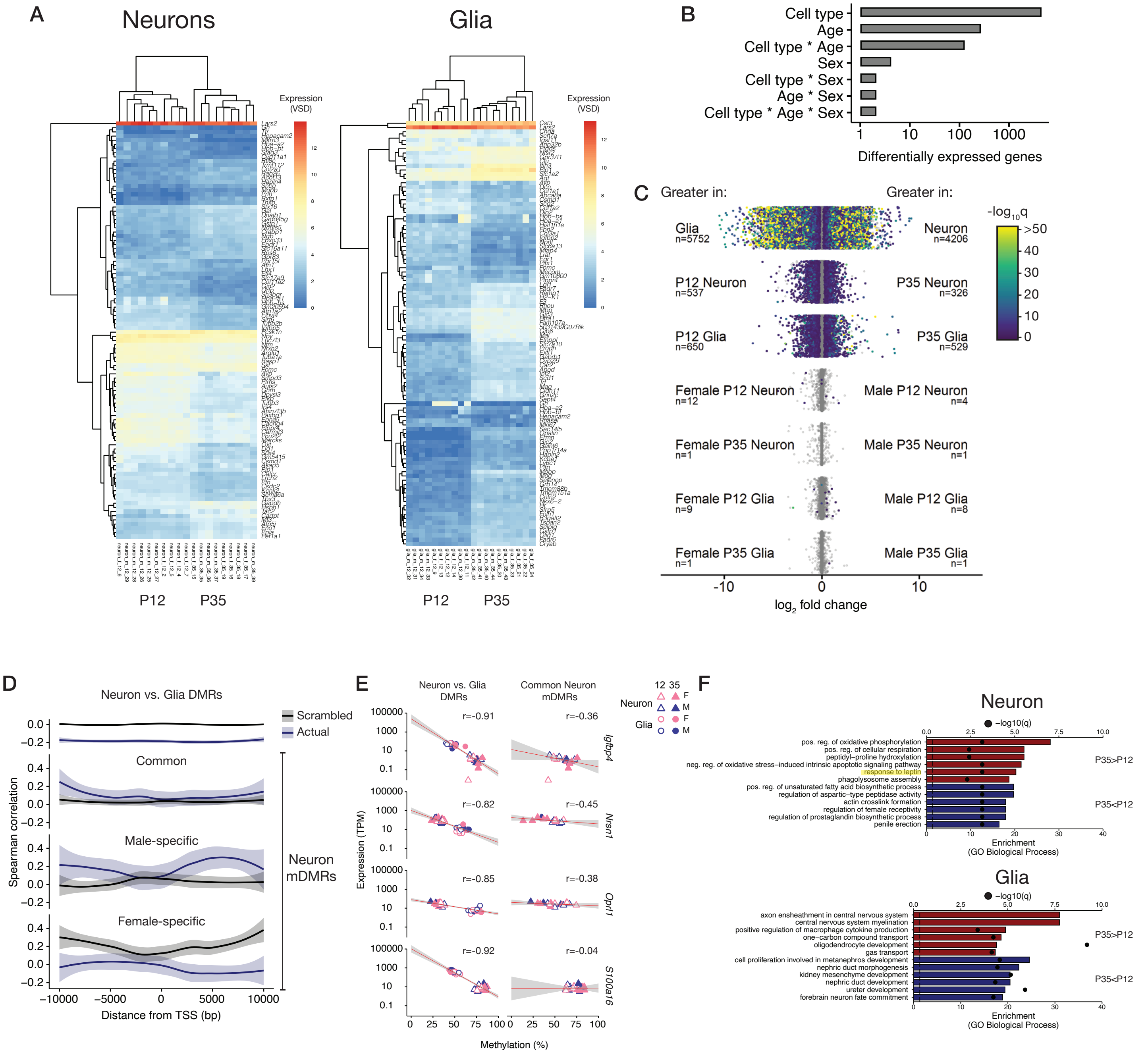

Figure S4 | Gene expression in ARH cells. A, Unsupervised clustering of the top 100 most variable genes (by SD) in neurons (left) and glia (right). Colour scale indicates variance stabilized expression levels. In both cases, samples cluster perfectly by age. **B,** Number of autosomal genes showing significant (FDR < 5%) main effects of cell type, age, and sex, and interactions between these factors. **C,** Scatter plots of differentially expressed genes for each of 7 pairwise comparisons. Fold-change values indicate greater expression in sample types listed on each side of the plots; numbers of significant DEGs for each are indicated. Points are colored by Benjamini-Hochberg-adjusted q-value. Genes not differentially expressed in that comparison are shown in gray. **D,** Spearman correlation between methylation level at TSS-flanking DMR loci and expression of associated genes relative to scrambled DMR-gene pairings. For neuron vs. glia DMRs near transcription start sites (TSS), gene expression is negatively correlated with methylation (top). No such correlation is evident at mDMRs (below). **E,** Scatter plots illustrating correlations between gene expression and promoter methylation at examples (from panel D) of neuron vs. glia DMRs and common neuron mDMRs. **F,** GO biological process results for P35 vs. P12 differentially expressed genes in neurons and glia. In each plot, enrichment values (bottom scale) are plotted as bars and -$\log_{10}$q values (top scale) are plotted as black dots.

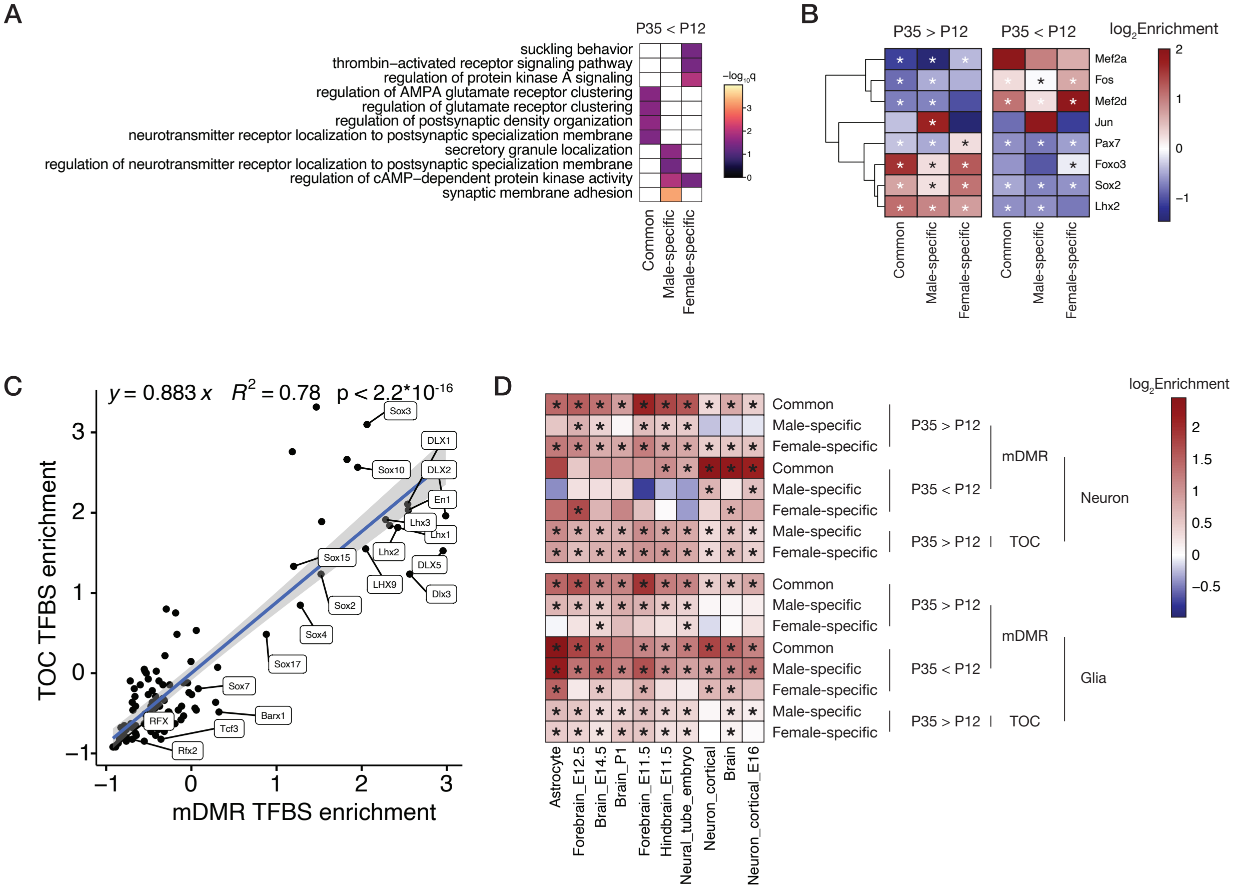

Figure S5 | Additional information associated with main Figure 3.
A, Gene ontology (GO) biological function analysis of genes associated with common and sex-specific P35<P12 neuron mDMRs. **B,** Enrichment of neural ChIP-Atlas transcription factor peaks in neuron mDMRs is consistent with the results based on TF motifs (Fig. 3b). **C,** As in males (Fig. 3c), scaled motif enrichment in female neuron TOCs is highly correlated with that at mDMRs ($p<2.2 \times 10^{-16}$). **D,** Enrichment of select EnhancerDB enhancers with mDMR and TOC features in neurons and glia. *Bonferroni-adjusted p-value < 0.05.

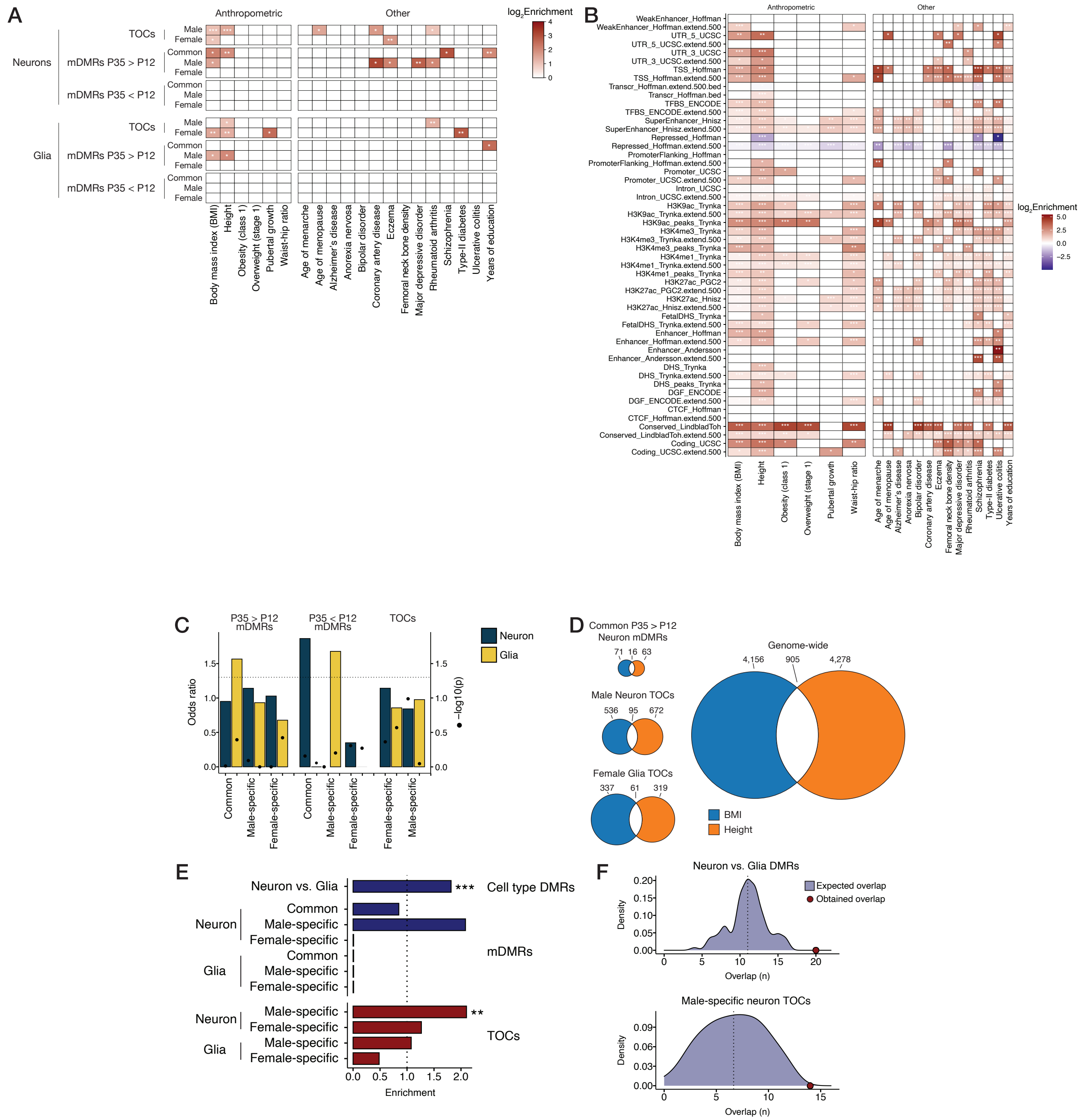

Figure S6 | Additional information associated with main Figure 4. A, Enrichment of explained heritability for BMI and height persist in mDMR and TOC loci when baseline epigenomic data **(B)** are included in the LDSC model. Only enrichment scores remaining significant after Benjamini-Hochberg correction are shown. **C,** Overlap between NHGHRI BMI and height index SNVs within mDMR and TOCs is not greater than expected by chance. Left X axis represents overlap ratio (plotted as bars). Odds ratio was calculated as the ratio of observed BMI:height SNV overlaps within a genomic feature to the number expected by random chance. Right Y axis represents p-value from Fisher's Exact Test (plotted as points; cutoff for p<0.05 indicated by dotted line). **D,** Venn diagrams illustrating overlap between BMI- and height-associated SNVs within select epigenomic features, and genome-wide. **E,** Enrichment of previously published ARH DMRs exhibiting sensitivity to early-life nutrition in DMR and TOC features relative to 100 sets of randomly generated control regions. *p<0.05, **p<0.01. **F,** Density plot showing overlap of previously published ARH DMRs with 100 sets of randomly generated control regions (Expected overlap) and DMR and TOC features. Dotted vertical line represents mean expected overlap. Enrichment in **E** is calculated as obtained overlap/expected overlap.

Supplementary tables

Table S1: Sample information and sequencing QC.
Table S2: Location and properties of maturational and sex DMRs in neurons and glia.
Table S3: Location and properties of Topped-Off Clusters (TOCs) in neurons and glia.
Table S4: Differential gene expression analysis by age and sex in neurons and glia.
Table S5: PhastCons scores and pairwise comparisons for mDMRs and TOCs.
Table S6: GWAS summary data information.
Table S7: Previously published developmental programming DMRs.
